# Hopanoid-free *Methylobacterium extorquens* DM4 overproduces carotenoids and has widespread growth impairment

**DOI:** 10.1371/journal.pone.0173323

**Published:** 2017-03-20

**Authors:** Alexander S. Bradley, Paige K. Swanson, Emilie E. L. Muller, Françoise Bringel, Sean M. Caroll, Ann Pearson, Stéphane Vuilleumier, Christopher J. Marx

**Affiliations:** 1 Department of Organismic and Evolutionary Biology, Harvard University, Cambridge, MA, United States of America; 2 Department of Earth and Planetary Sciences, Washington University in St Louis, St Louis, MO, United States of America; 3 Equipe Adaptations et interactions microbiennes, Université de Strasbourg, UMR 7156 UNISTRA–CNRS Génétique Moléculaire, Génomique, Microbiologie, Strasbourg, France; 4 Department of Earth and Planetary Sciences, Harvard University, Cambridge, MA, United States of America; 5 Department of Biological Sciences, University of Idaho, Moscow, ID, United States of America; 6 Institute for Bioinformatics and Evolutionary Studies, University of Idaho, Moscow, ID, United States of America; 7 Center for Modeling Complex Interactions, University of Idaho, Moscow, ID, United States of America; University of Münster, GERMANY

## Abstract

Hopanoids are sterol-like membrane lipids widely used as geochemical proxies for bacteria. Currently, the physiological role of hopanoids is not well understood, and this represents one of the major limitations in interpreting the significance of their presence in ancient or contemporary sediments. Previous analyses of mutants lacking hopanoids in a range of bacteria have revealed a range of phenotypes under normal growth conditions, but with most having at least an increased sensitivity to toxins and osmotic stress. We employed hopanoid-free strains of *Methylobacterium extorquens* DM4, uncovering severe growth defects relative to the wild-type under many tested conditions, including normal growth conditions without additional stressors. Mutants overproduce carotenoids–the other major isoprenoid product of this strain–and show an altered fatty acid profile, pronounced flocculation in liquid media, and lower growth yields than for the wild-type strain. The flocculation phenotype can be mitigated by addition of cellulase to the medium, suggesting a link between the function of hopanoids and the secretion of cellulose in *M*. *extorquens* DM4. On solid media, colonies of the hopanoid-free mutant strain were smaller than wild-type, and were more sensitive to osmotic or pH stress, as well as to a variety of toxins. The results for *M*. *extorquens* DM4 are consistent with the hypothesis that hopanoids are important for membrane fluidity and lipid packing, but also indicate that the specific physiological processes that require hopanoids vary across bacterial lineages. Our work provides further support to emerging observations that the role of hopanoids in membrane robustness and barrier function may be important across lineages, possibly mediated through an interaction with lipid A in the outer membrane.

## Introduction

Hopanoids are pentacyclic triterpenoids present in some bacteria that are ubiquitous in sediments and sedimentary rocks. These types of molecules are among the best documented organic geochemical biomarkers in the rock record, and have been detected in rocks at least 1.7 billion years old [1]. Hopanoids are widely used by geochemists as general indicators of bacterial presence in ancient environments [2].

Previous studies of the physiological role and regulation of hopanoid production have revealed diverse results across taxa. *Streptomyces coelicolor* synthesizes hopanoids only during the formation of aerial hyphae, possibly to decrease the permeability of water across the membrane [3], whereas *Streptomyces scabies* produced hopanoids during submerged growth [4]. In the root nodule-colonizing bacterium *Frankia alni*, hopanoids were over-expressed in nodules [5], and enriched in nitrogen-fixing vesicles in *Frankia* spp., where they may function as a permeability barrier to oxygen [6]. A permeability role for hopanoids was also inferred in the alphaproteobacterium *Zymomonas mobilis*, which displayed an increased sensitivity to ethanol concentrations when hopanoid production was diminished by the addition of azasqualene, which inhibits the key enzyme squalene-hopene cyclase (SHC) that catalyzes the synthesis of hopanoids [7]. In the gammaproteobacterium *Frateuria aurantia*, hopanoid production increased with growth temperature [8]. Similarly, in the Firmicute *Alicyclobacillus* (formerly *Bacillus) acidocaldarius*, the abundance of side-chain containing hopanoids is positively correlated with growth at increased temperature or decreased pH [9]. The authors of the latter study hypothesized that hopanoids help overcome the expected increased permeability and decreased stability of biological membranes under these conditions.

Hopanoid-free mutants have yielded further insight into hopanoid function. A hopanoid-free mutant of *S*. *scabies* showed no growth phenotype under normal conditions, formed aerial hyphae, and did not differ from wild-type (WT) in its response to oxidative stress, osmotic stress, or tolerance of ethanol, high temperature, or pH stress [4]. A hopanoid-free mutant of the betaproteobacterium *Burkholderia cenocepacia* displayed no growth defect at neutral pH, but was sensitive to pH stress, antibiotics, and detergents [10]. In *Burkholderia multivorans*, a role for hopanoids in multiple antimicrobial resistance was suggested, based on increased membrane permeability in the mutant [11], and susceptibility to polymixin antibiotics in the presence of the isoprenoid synthesis inhibitor fosmidomycin [12]. Growth of a hopanoid-free mutant of the alphaproteobacterium *Rhodopseudomonas palustris* was indistinguishable from WT under standard conditions, but exhibited sensitivity to pH shock, bile salts, and antibiotics [13]. A *Bradyrhizobium* strain, another alphaproteobacterium, was found to covalently link hopanoids to lipid A in the outer leaflet of the outer membrane, and the hopanoid-free mutant shows increased sensitivity to stress [14]. Furthermore, in *B*. *diazoefficiens* hopanoids are apparently essential for growth [15]. In the cyanobacterium *Nostoc punctiforme*, a hopanoid-free mutant grew more poorly than WT under temperature stress, but better at low temperatures [16]. These results across organisms suggest that hopanoids play a role in membrane rigidity and integrity, with a pronounced link with the outer leaflet of the outer membrane. This is supported by biophysical data suggesting that hopanoids are associated with lipid A in the outer leaflet of the outer membrane, where they interact with glycolipids [17].

One type of microbial metabolism that has been little explored with regard to hopanoid function is methylotrophy. During growth on single-carbon compounds like methanol, methylotrophs generally oxidize these substrates to formaldehyde in the periplasm, and the formaldehyde is then utilized in the cytoplasm. This partitioning of the production and use of the toxic intermediate formaldehyde may make it necessary to maintain a high level of inner and outer membrane integrity. Members of the alphaproteobacterial genus *Methylobacterium* have long been known to produce hopanoids. These hopanoids include C_31_ hopanoids containing an additional methyl group at C2 [18,19], as well as those with side chains added through the successive action of enzymes encoded by *hpnG* and *hpnH* [20]. The first indication that the loss of hopanoids causes growth defects in *Methylobacterium* arose from having identified a *M*. *extorquens* DM4 isolate with a minitransposon insertion into *shc* that caused it to lose the ability to grow on dichloromethane (DCM) [21]. The mutant strain was determined to be hopanoid-free [20] [21]. Recently, a hopanoid-free *M*. *extorquens* PA1 mutant was used in a biophysical study that reported this strain to have lowered membrane order [17]. That work established that in *Methylobacterium* hopanoids are preferentially localized to the outer membrane, where they interact with lipid A. The only phenotypic analysis of this mutant, however, was to look at sensitivity to Triton X-100, which was increased by 1000-fold [17]. Here we characterize the changes in lipid content for the previously-isolated *M*. *extorquens* DM4 *shc*::miniTn*5* strain (hereafter *shc* mutant) [21] and characterize a wide variety of growth defects.

## Materials and methods

### Media and growth conditions

*M*. *extorquens* DM4 WT strain (DSMZ 6343) and its hopanoid-free, *shc* mutant 41C5 [21] were grown in minimal medium at 30°C as previously described [22,23]. For liquid growth, carbon sources were added alone or in combination at the following concentrations (unless stated otherwise): succinate (3.5 mM), methanol (15 mM), DCM (10 mM) or betaine (*i*.*e*., trimethylglycine, 10 mM). On 1.8% agar plates, substrate concentrations were increased to 15 mM succinate, or 125 mM methanol. Where the pH of the medium was adjusted, it was via addition of HCl or KOH. In some experiments, which we specifically note below, we utilized an optimized version of Hypho medium described in [24], that we refer to as MPIPES. Where utilized, bile salts were added to a concentration of 1.5%. Cell culture was grown aerobically to mid-exponential phase in up to 50 mL batches for harvest for lipid analysis. Growth rate analyses were performed in 48-well plates (Costar) containing 640 μL of medium per well in an automated system situated in a warm room (30°C, 80% humidity) that consisted of a shaking plate tower (Liconic), a TwisterII microplate handler (Caliper), and a Wallac Victor2 plate reader (Perkin Elmer) operated by the software program Clarity [25]. Plates were shaken at a rate of 650 rpm and optical density was measured hourly at 600 nm. Growth rates for DCM were determined in 250 mM Erlenmeyer flasks containing 50 mL medium supplied with 32 μL of pure liquid DCM (Fluka) with gas-tight screwcaps equipped with miniert valves (Sulpelco). Tolerance to various stresses was evaluated by triplicate measures of the sizes of growth inhibition halos on plates as described previously [21]. *Escherichia coli* strains were grown at 37°C in Luria-Bertani broth. Antibiotics were added for selection at the following final concentrations: ampicillin 50 μg/mL, chloramphenicol 20 μg/mL, rifamycin 50 μg/mL, streptomycin 35 μg/mL, and tetracycline 10 μg/mL.

Liquid cultures were supplemented with an exogenous solution of cellulase enzyme to reduce cell clumping and permit accurate calculations of growth rates from a time series of optical density measurements. The cellulase solution was prepared by diluting 2 g of purified *Aspergillus niger* cellulase (Sigma-Aldrich, St. Louis, MO) into 10 mL of molecular grade water. To remove chemical impurities, the cellulase was loaded into a pre-wetted 10K MWCO Slide-A-Lyzer cassette (Thermo Fisher Scientific, Rockford, IL) and dialyzed twice for 1.5 hours in 4 L of deionized water at 4°C; longer dialysis times were found to impair the integrity of the dialysis cassette membrane. To ensure sterility, the dialyzed cellulase was passed through a 0.2 μm syringe filter (VWR) and diluted with sterile molecular grade water to a final concentration of 10 mg/mL or 20 mg/mL, as quantified on a ND-1000 spectrophotometer (Thermo). The final solution was found to be stable at 4°C for at least several months. Cellulase added to approximately 0.1 mg/mL in growing cultures eliminated most of the noise in optical density measurements caused by clumping, yet had no effect on cellular growth rate or yield (see below).

### Lipid extraction and analysis

Lipids were extracted from *M*. *extorquens* DM4 cells following the method of Bligh and Dyer [26], with phosphate-buffered saline (PBS) substituted for the aqueous portion of the mixture to improve extraction of intact polar lipids (IPLs). Total lipid extracts were dried under a stream of N_2_ and stored at -20°C in the dark until analysis.

Hopanoids were analyzed by subjecting the total lipid extract to oxidation by periodic acid. Aldehydes produced by this process were subsequently reduced to alcohols with sodium borohydride (NaBH_4_) after the method of Rohmer *et al*. [27]. Alcohols were derivatized with *N*,*O*-bis(trimethylsilyl)trifluoro-acetamide (BSTFA) and 1% trimethylchlorosilane (TMCS) in the presence of pyridine, and analyzed via GC-MS on an Agilent 6890 gas chromatograph coupled to an Agilent 5973 quadrupole MS operating in full scan mode between *m*/*z* 50 and 750. In both LC-MS and GC-MS analyses, hopanoids were identified by comparison of retention times and mass spectra to previously published information [28–30], or to samples with established hopanoid compositions. Hopanoid quantification was via GC-MS relative to an internal standard (epiandrosterone, Sigma). Squalene was also present in some samples and was quantified relative to an internal standard (squalane, Aldrich).

The fatty acid content of *M*. *extorquens* DM4 strains was determined by converting fatty acids to fatty acid methyl esters (FAMEs) in methanolic boron trifluoride (BF_3_/MeOH kit, Sigma) for 15 minutes at 70°C, followed by quenching with 2 mL of DCM-extracted deionized water. Transesterified lipids were extracted from this mixture with hexane, and dried over Na_2_SO_4_. Analysis occurred via GC-MS on the Agilent system described above, and FAMEs were quantified relative to an internal standard (lignoceric acid methyl ester; Sigma-Aldrich).

Total carotenoid content was quantified by suspending Bligh-Dyer lipid extractions in 250 μL of chloroform and determining the absorbance of the extract at 512 nm utilizing a Nanodrop 5000c spectrophotometer. Absorbance was converted to carotenoid concentration by comparison to a carotenoid standard calibration curve and normalization to cell number.

### Construction of a plasmid to complement the *shc* mutant

A plasmid for squalene-hopene cyclase expression was obtained by first amplifying the *shc* gene from *M*. *extorquens* DM4 with primers that incorporated the ribosomal binding site from *fae* [31], a highly-expressed protein that catalyzes the first step of formaldehyde oxidation [32]. The resulting product was cloned into pCR-BluntII TOPO (Invitrogen), yielding the plasmid pAB169. The insert was then excised via *Xba*I/*Kpn*I and ligated into the corresponding sites of the plasmid pCM62 [33], resulting in pAB170. pAB170 was mated from *E*. *coli* (10-beta, New England Biolabs) into the hopanoid-free mutant via tri-parental mating with *M*. *extorquens* DM4 and *E*. *coli* strain pRK2073 [34], resulting in strain CM3926. After analysis of the plasmid-complemented strain, a second plasmid, pPS10, was designed to complement the mutant by reinsertion of the native *shc* gene onto the chromosome in place of the miniTn*5* disruption. pPS10 was constructed by amplifying the *shc* gene from *M*. *extorquens* DM4 and cloned into pCM433 [35]. pPS10 was integrated into the *shc* mutant via tri-parental mating with *E*. *coli* strain pRK2073, resulting in the double-crossover chromosomal *shc* restoration strain CM4201.

### Toxicity disk assays

Toxicity assays were performed as described previously [21]. Briefly, on exactly 20 mL of solidified mineral medium containing 1.5% agar containing 10 mM methanol, 2.5 mM succinate, and 10 μg/mL tetracycline base, an overlay was added of precisely 7 mL of the same medium containing 0.7% agar to which a suspension of the strain to be investigated, grown in the same medium, was added to a final OD_600_ of 0.2. One 6 mm filter disk was added to each plate and spotted with 5 μL of a solution of the toxicant to be investigated, and plates were incubated face-up for 4 days at 30°C before measuring the diameter of growth inhibition.

### Illumina resequencing of mutant genome

Cells of the *shc* mutant were grown to an optical density of 0.2 and harvested by centrifugation. Genomic DNA was extracted using a Wizard Genomic DNA purification kit (Promega) and checked for purity using a Nanodrop 5000c spectrophotometer. Genomic libraries were prepared at the Genome Technology Access Center at Washington University in St. Louis, followed by paired end (2x101) sequencing on an Illumina HiSeq 2000. Sequence files were compared to the *M*. *extorquens* DM4 reference genome using *breseq* [36].

## Results

### The *shc* mutant strain of *M*. *extorquens* DM4 lacks hopanoids

**Fig 1:**
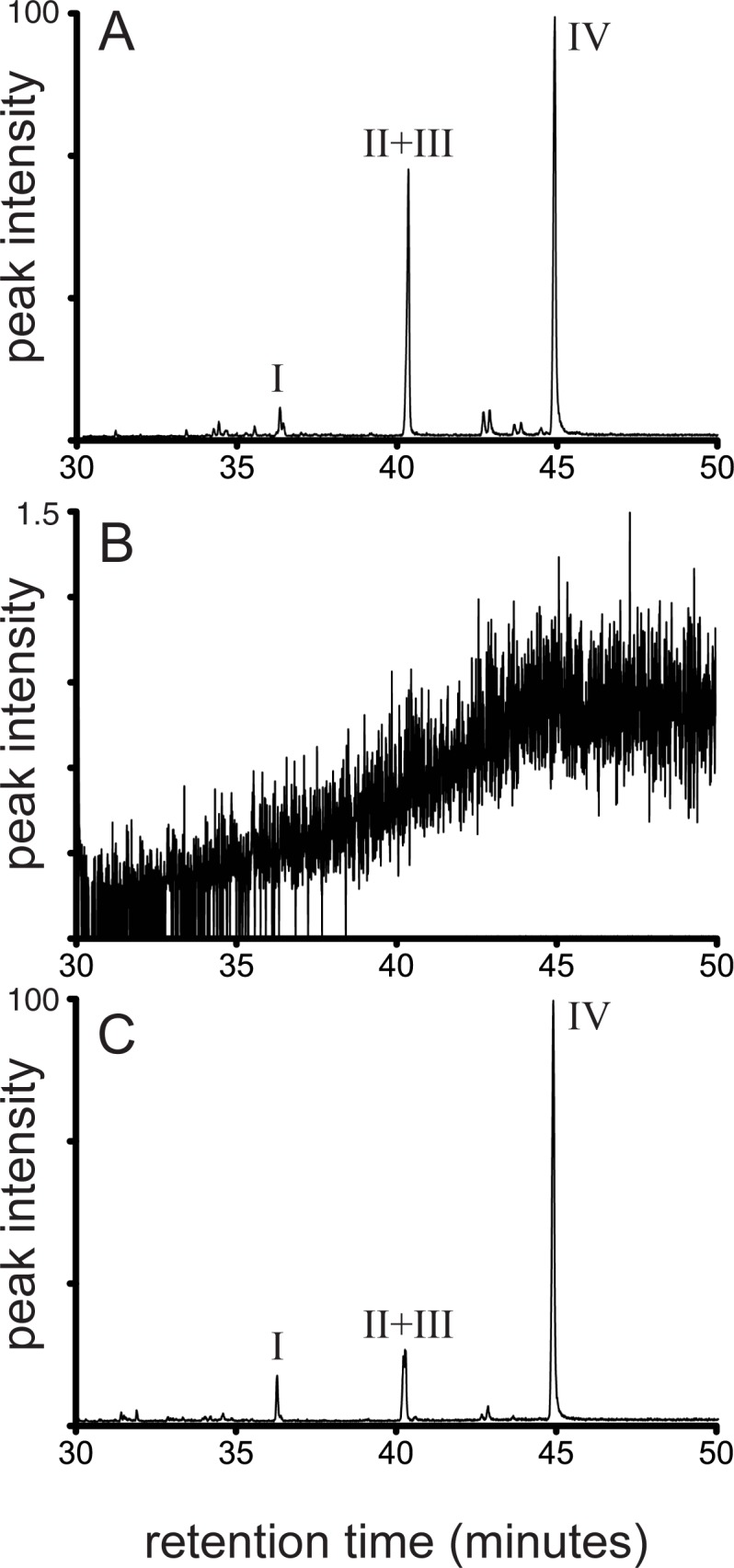
GC-MS *m/z =* 191 trace of total lipid extracts of *Methylobacterium extorquens* DM4. A) wild-type: hopanoid peaks shown are diploptene (I) and diplopterol (II) + methyldiplopterol (III). B) hopanoid-free *shc* mutant showing absence of hopanoids C) *shc* mutant complemented with an *shc*-expressing plasmid showed a product accumulation similar to WT. Roman numerals refer to structures given in S1 Fig.

As a first stage in characterization of the *M*. *extorquens* DM4 *shc* mutant, its lipid content was compared to WT. While the GC-MS trace of the lipid extracts analysis for WT showed the presence of several hopanoid structures (Fig 1; Table 1), the *shc* mutant did not accumulate hopanoids (Fig 1). Although hopanoid structures were absent in *shc* mutant, the hopanoid precursor compound squalene was observed. The concentration of squalene in the mutant was lower than that of hopanoids in the WT cells (Table 1). Complementing the *shc* mutant with a plasmid expressing *shc* from the *E*. *coli lac* promoter (which is constitutively expressed at a low level in *M*. *extorquens* [31] recovered the hopanoid structures observed in the WT. The level of hopanoid accumulation in the complemented mutant was not to the same concentration as in the WT, and the level of squalene remained elevated (Table 1).

**Table 1:** Lipid content of *M*. *extorquens* DM4 strains grown on succinate (μg/mg dry wt ± 1σ). Homohopanoids are defined as hopanoids containing a side chain.

Lipid	wild- type	*shc* mutant	*shc* mutant + *shc* plasmid
squalene	nd	0.22 ± 0.09	0.61 ± 0.10
diploptene	0.11 ± 0.07	nd	0.10 ± 0.02
diplopterols	4.83 ± 0.41	nd	1.05 ± 0.01
% methylated	61.80%	nd	34.80%
homohopanoids	2.15 ± 0.18	nd	0.64 ± 0.01
carotenoids	0.03 ± 0.01	0.36 ± 0.03	n/a

nd = not detected, n/a = not measured

### The *shc* mutant strain of *M*. *extorquens* DM4 has an altered fatty acid profile

**Fig 2:**
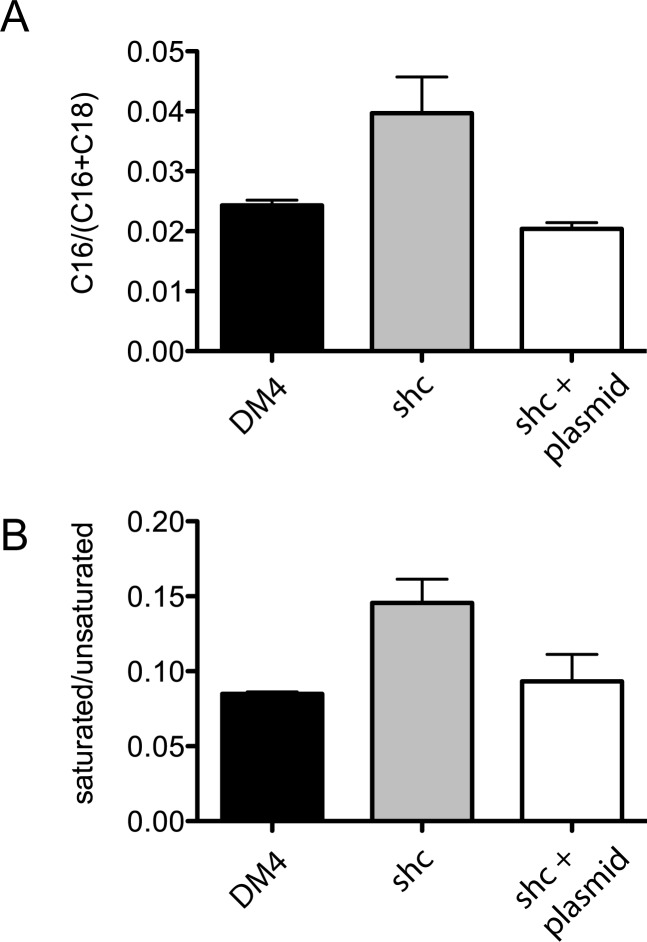
Fatty acid production of wild-type and *shc* mutant strain grown on succinate. A) Ratio of total C_16_ fatty acids to the sum of C_16_ + C_18_ fatty acids. B) Ratio of saturated/unsaturated fatty acids. Complementation plasmid carries the *shc* gene.

Both WT and *shc* mutant strains contained C_16_ and C_18_ saturated and mono-unsaturated fatty acids, with C_18_ mono-unsaturated fatty acid as the overwhelmingly most abundant fatty acid (> 85% of total fatty acids in all strains). The IPL distributions suggested that the hopanoid-free mutant strain produced relatively more C_16_ fatty acid than C_18_ fatty acid, as compared to the WT. This was confirmed by analysis of the fatty acid content. The mutant strain had a slightly higher proportion of C_16_ fatty acids overall (Fig 2A), and about twice the proportion of saturated fatty acids as the WT (Fig 2B).

### The *shc* mutant strain of *M*. *extorquens* DM4 overproduces carotenoids

**Fig 3:**
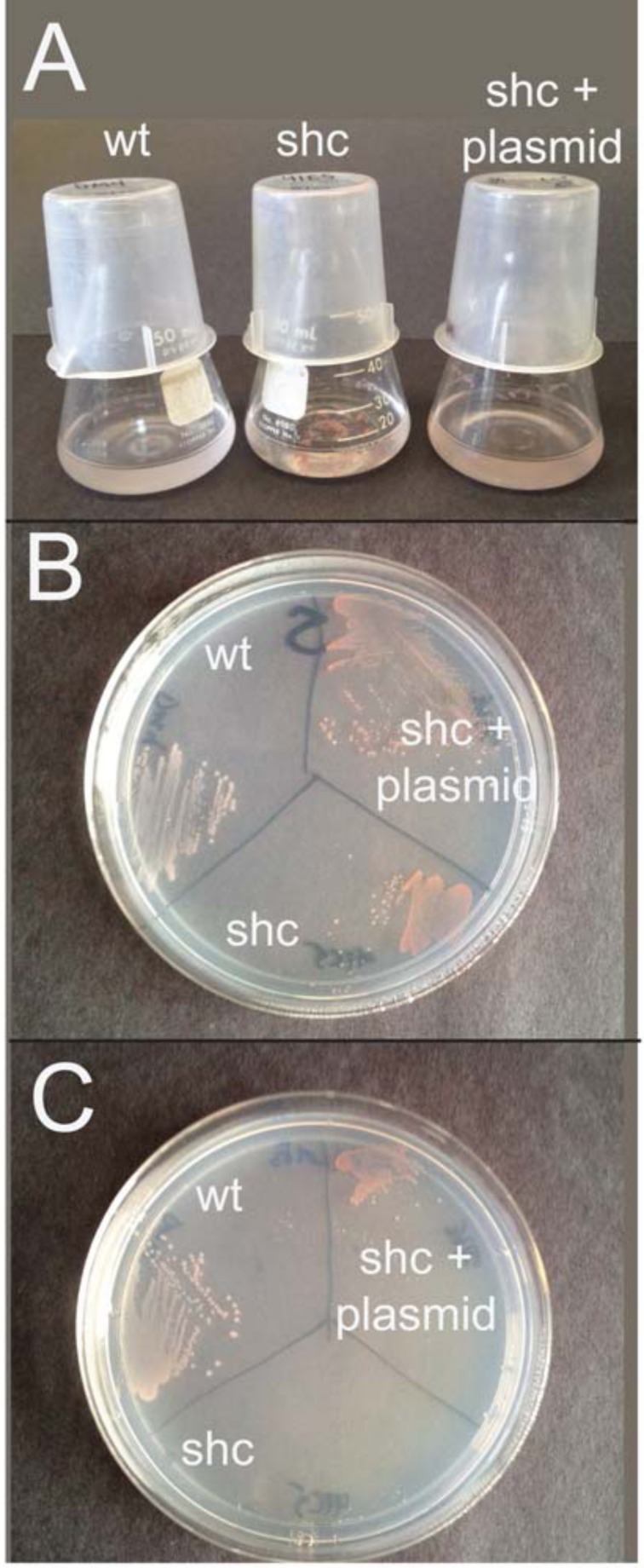
Growth of wild type and mutant. A) Growth on succinate in liquid media of WT DM4, *shc* mutant (showing clumping), and complemented *shc* mutant. B) Growth on succinate plates showing over-accumulation of pigmentation in *shc* and complemented *shc* relative to WT strain. C) Growth on plate containing succinate as growth substrate and 1 mM formaldehyde. WT strain and complemented mutant grow, but *shc* mutant is unable to grow. Complementation plasmid carries the *shc* gene.

Given that hopanoids and carotenoid pigments are both derived from isoprenoid precursors, we examined the carotenoid content of the WT and mutant strains. Visually, the *shc* mutant was substantially darker pink than WT (Fig 3). When quantified spectrophotometrically following Bligh-Dyer extraction, this difference was revealed to result from a greater than 10x increase in carotenoid concentration compared to WT strain (Table 1).

### Hopanoid-free mutant strain is hypersensitive to a wide range of stressors

In order to determine whether the *shc* mutant renders *M*. *extorquens* DM4 sensitive to external stresses, we examined the inhibitory effect of a variety of agents relative to WT. It should be noted that our complementation plasmid contains the *tetA* tetracycline resistance pump, which is well know for affecting sensitivity to other factors [37]. However, we needed to use this plasmid because the transposon already introduced kanamycin resistance into the chromosome. Growth of the hopanoid-free mutant was inhibited on agar plates containing 1 mM formaldehyde, while WT strain and complemented strains grew normally (Fig 3). Similar results were obtained during growth on bile salts (1.5%), although in this case the complemented strain did not recover the ability to grow. We also examined the zone of growth inhibition imparted by a number of potential toxins and antibiotics (Table 2). Many such compounds affected the *shc* mutant more than the WT, consistent with the phenotype predicted by Saenz *et al*. [17]. In some instances, these effects were only partially relieved by complementation–for example rhodamine toxicity is only slightly alleviated through recovery of hopanoids by the *shc* plasmid.

**Table 2:** Sensitivity of strains to toxic compounds in disk diffusion assays.

Toxicant ^a^		Relative growth inhibition ^b^		Comments
	*shc* mutant	*shc* mutant+vector	*shc* mutant+*shc* plasmid	
SDS 347 mM	2.44	1.53	1.08	Detergent, membrane disruption
Rhodamine 210 mM	2.08	1.86	1.71	Genotoxicity
Novobiocin 163 mM	1.91	1.23	0.97	DNA gyrase inhibitor
Chloramphenicol 62 mM	1.83	1.6	0.93	Protein synthesis inhibitor
Ethidium bromide 1.3 mM	1.72	1.1	0.99	Genotoxicity
Tetracycline HCl 45 mM	1.56	0	0	Protein synthesis inhibitor; plasmids confer TetR
Formaldehyde 12 M	1.56	1.09	1.12	Genotoxicity
Methylglyoxal 5.5 M	1.4	1.32	1.21	Genotoxicity
Triclosan 35 mM	1.39	1.05	1.06	Fatty acid synthesis inhibitor
Streptomycin 43 mM	1.28	1.28	1.1	Protein synthesis inhibitor
Ampicillin 286 mM	1.27	1.01	0.97	Peptidoglycan biosynthesis inhibitor
Hydrogen peroxide 10 M	1.22	1.11	1.09	Oxidative stress
Crystal violet 56 mM	1.12	1.08	0.97	Genotoxicity

^a^ 70% or 100% ethanol, 100% DMSO, 0.3M NaOH or 3 M sodium acetate were used to solubilize toxic compouds and had no growth inhibition when used alone. Bacitracine (14 mM), cycloheximide (180 mM), trimethoprim (35 mM), Cr^3+^, Pb^2+^, Co^2+^, Zn^2+^, Cd^2+^, Cu^2+^ (2 M), As^3+^ (13 mM) had no effect on growth under the used experimental conditions with both strains tested.

^b^ The measured diameter of growth inhibition observed with the wild-type strain *M*. *extorquens* DM4 in at least duplicate assays was set to 1. Numbers are reported as averages of replicates (n = 2 or 3).

### Hopanoid-free mutant strain grows poorly in liquid media and flocculates due to cellulose production

**Fig 4:**
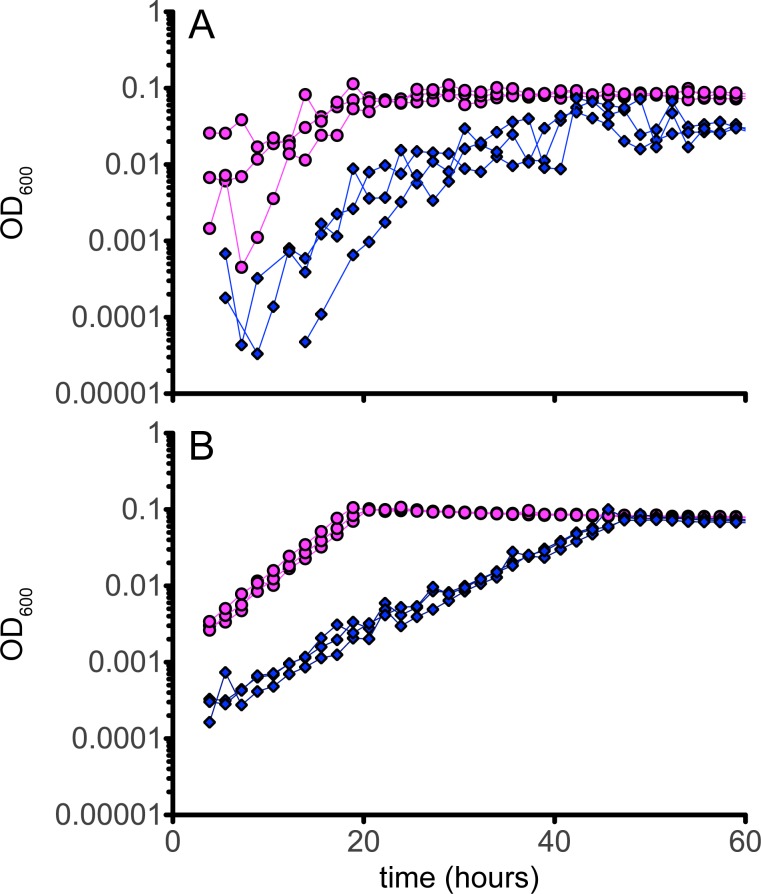
Growth curves of *M*. *extorquens* DM4 strains with succinate as the growth substrate. The colors represent triplicate experiments for A) wild type (pink circle) and mutant (blue diamond) strains without cellulase addition, and B) wild type (pink circle) and mutant (blue diamond) strains with cellulase addition.

The hopanoid-free *shc* mutant strain was able to grow on both single-carbon and multi-carbon substrates such as methanol, formate, succinate, acetate, ethanol, glycerol, pyruvate, and betaine on agar plates, but was unable to grow on DCM in liquid culture, as reported previously [21]. Regardless of the carbon substrate, however, growth of the mutant on liquid media showed a defect in comparison to the WT, with low yields (as measured by total dry weight) and pronounced flocculation (Figs 3 and 4).

**Fig 5:**
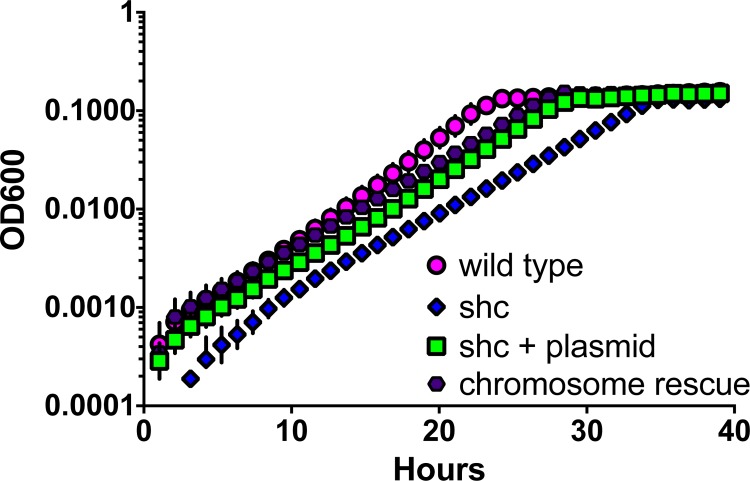
Growth curves of *M*. *extorquens* DM4 strains in optimized media. Succinate is the growth substrate. The colors represent 6x replicate experiments for wild-type (dark pink), *shc* mutant (dark blue), *shc* mutant with *shc* plasmid (green square), and *shc* mutant that has been rescued with chromosomal *shc* insertion (purple hexagon).

We utilized an automated, robotic growth analysis system to determine growth rates for the mutant strain on C_1_ and multi-C substrates. Due to the flocculation in the *shc* mutant, however, it was difficult to determine a growth rate for this strain. Other reports had demonstrated that minor flocculation in *M*. *extorquens* can be ameliorated through removal of genes required for cellulose synthesis, suggesting a role of cellulose in biofilm formation [24]. This suggested that the severe flocculation in the *shc* mutant might be similarly ameliorated, and when the media was supplemented with purified cellulase, a large decrease in flocculation occurred. This allowed accurate determination of growth rates for wild-type and mutant strains. During growth on succinate in media amended with cellulase, the WT and complemented strains doubled in 2.6 hours, while the *shc* mutant had a doubling time of 4.9 hours. Similarly, on methanol plus cellulase the WT and complemented strains doubled in 2.9 hours, while the *shc* mutant had a doubling time 5.6 hours (Table 3), with final optical densities of the mutants indistinguishable from that of the wild type. The growth defect was also severe on formate, where the WT doubling time was 4.5 hours, while the mutant doubling time was 7.2 hours. This analysis confirms a substantial, generic growth defect for the mutant strain, in addition to the observed clumping (Fig 5).

**Table 3:** Doubling times (hours ± 1σ) of *M*. *extorquens* DM4 strains grown on various substrates^a^. Mean of three replicates per strain/substrate combination.

Substrate	WT	*shc* mutant	mutant + *shc* plasmid	WT + vector
succinate	2.6 ± 0.1	4.9 ± 0.3	3.1 ± 0.1	2.5 ± 0.1
methanol	2.9 ± 0.1	5.6 ± 0.5	3.4 ± 0.1	2.8 ± 0.1
DCM	9.0^b^	no growth^b^	6.0^b^	7.5^b^
acetate	4.5 ± 0.8	8.2 ± 0.4	7.6 ± 1.2	4.8 ± 0.2
ethanol	5.1 ± 0.5	5.3 ± 0.7	4.1 ± 0.2	3.6 ± 0.1
formate	4.5 ± 0.5	7.2 ± 1.3	4.9 ± 0.2	4.1 ± 0.3
pyruvate	2.8 ± 0.1	5.2 ± 0.1	3.6 ± 0.1	3.1 ± 0.2
betaine	4.0 ± 0.5	5.2 ± 1.0	3.9 ± 0.3	3.5 ± 0.2

^a^ Growth under conditions described in Lee *et al*., 2009 except were noted

^b^ Growth under conditions described in Muller *et al*., 2011 with tetracycline (10 μg/mL) for plasmid maintenance as required

### Hopanoid-free mutant strain grows poorly under pH stress

**Fig 6:**
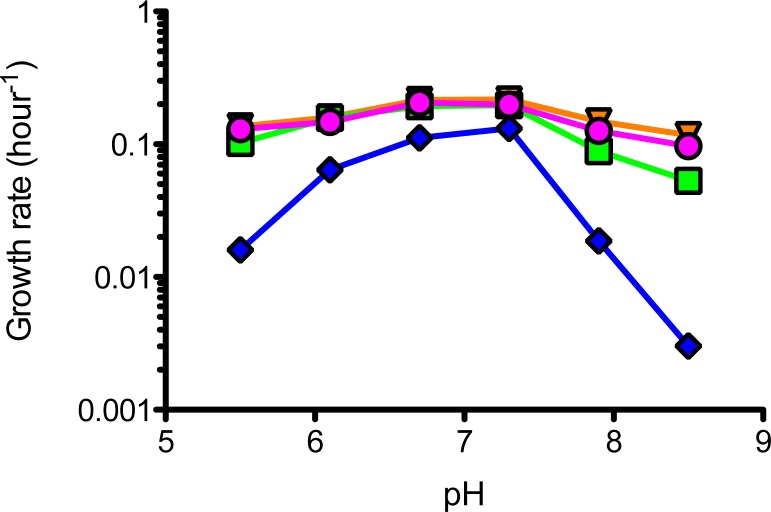
Doubling times of *M*. *extorquens* DM4 wild-type and mutant strains as a function of medium pH. The colors represent triplicate experiments for wild-type (pink circles), wild-type with empty vector (orange triangles), *shc* mutant (blue diamonds), *shc* mutant with *shc* plasmid (green square). Growth rates measured in the presence of cellulase.

We grew the WT and mutant strains of *M*. *extorquens* DM4 in liquid media amended with cellulase, with succinate as a growth substrate under a range of pH conditions. We found that at near-neutral pH, the growth rate of the mutant strain lagged behind both the WT and the complemented strains. When grown in media where the pH had been adjusted to more extreme alkaline or acidic values, the growth rate of the mutant strain became markedly worse, with growth rates dropping to levels that were approximately 10% that of the WT and complemented strains (Fig 6).

### Partial complementation reveals that the hopanoid-free mutant strain accumulated another mutation

A plasmid expressing *shc* was generated to complement the mutant phenotypes observed. The growth defects observed for the *shc* mutant were partially reversed when complemented (Table 3). This included the previously reported [21] growth defect of the *shc* mutant with DCM as the sole carbon source. The intermediate growth phenotypes of the complemented strain may either be due to an incomplete return to the lipid profile of wild-type (see above) and/or toxicity from expressing *shc* from a multi-copy plasmid, polar effects of the transposon insertion, or to the accumulation of secondary mutations that were acquired during the mutagenesis of the WT.

To distinguish between these three possibilities, we compared the growth of the WT and mutant strain to a second complemented mutant strain in which the *shc* gene was inserted into the chromosome of *M*. *extorquens*, thereby restoring the *shc* locus to its wild-type state (Table 4). These experiments were performed in media optimized for *Methylobacterium* [24]. Results demonstrated that even with insertion of *shc* into the site of Tn*5* disruption, full recovery of the WT phenotype was not achieved.

**Table 4:** Doubling times (hours ± 1σ) of *M*. *extorquens* DM4 strains on succinate in optimized media. Mean of three replicates per strain/substrate combination.

Strain	Doubling time
WT	2.7 ± 0.05
*shc* mutant	3.7 ± 0.10
*shc* mutant + *shc* plasmid	3.3 ± 0.04
*shc* mutant + chromosome rescue	3.4 ± 0.08
WT + vector	2.0 ± 0.01

After observation that neither complementation of *shc* on a plasmid nor insertion of *shc* back onto the chromosome resulted in full recovery of the wild-type phenotype, we hypothesized that mutations had occurred during the mutagenesis of the WT to recover the *shc* mutant. We sequenced the genome of the *shc* mutant to detect these mutations. This analysis revealed only a single confirmed mutation, a single nucleotide polymorphism (CCT → CCC) at site 216230, which results in a synonymous mutation in a proline of a gene encoding a sulfonate transport system binding protein (METDI0211).

## Discussion

Although hopanoids are not required for the growth of *M*. *extorquens* DM4, their absence imposes myriad constraints on growth. This result is similar to that observed in two other alphaproteobacteria, *Bradyrhizobium* [14], and *R*. *palustris* [13], which had *shc* mutants that display phenotypes under osmotic and pH stress. In *M*. *extorquens* a growth rate defect is observed even under optimal conditions, but final cellular densities of the mutant are as great as the WT. This suggests that the general importance of hopanoids is most significant under particular environmental conditions, which may relate to their physiological function. While this function is unknown, it likely relates to membrane permeability, and perhaps to cell division [38]. A recent suggestion, based on data from *M*. *extorquens* PA1, is that hopanoids order the outer membrane allowing for critical functions such as multidrug efflux [17]. Some hopanoid structures have been suggested to have a more particular function: for example, methylation at the C2 position has been related to membrane rigidification [39] and correlated with plant-microbe associations [38] [40]. Accumulation of high amounts of methyldiplopterol in *Methylobacterium* strains–a plant-associated microbe [41]–is consistent with this hypothesis. Deletion of the gene responsible for hopanoid methylation results in accumulation of enhanced amounts of tetrahymanol in *R*. *palustris* TIE-1 [42]. *Methylobacterium* does not accumulate tetrahymanol, a hopanoid-derived lipid that is produced in some hopanoid-containing bacteria by an additional enzymatic step [43].

Despite the fact that the *shc* mutant was highly sensitive to formaldehyde, and that growth with methanol leads to formaldehyde production, the relative growth defect of the *shc* mutant was similar for growth on methanol as on succinate, or almost any of the other compounds tested. Except for DCM, where it is essential to have *shc*, and ethanol, where there was no significant growth difference, the *shc* mutant grew 50–100% slower on all other substrates tested. We had expected to see a defect on methanol or betaine in particular, as these compounds lead to formaldehyde production, but this result suggests that either the formaldehyde level during methanol (or betaine) growth was sufficiently low to have less effect than 1 mM exogenous formaldehyde, or that the growth substrate may alter other aspects of sensitivity to formaldehyde. One possible explanation is that the documented induction in formaldehyde oxidation capacity (e.g., the tetrahydromethanopterin pathway) during growth on methanol is sufficient to buffer the effects of formaldehyde production [44,45].

Although the particulars of the selective environments of hopanoid-containing microbes clearly differ, there may still be some generalities as to their immediate biochemical role. Altered susceptibility to bile salts was interpreted to indicate that membrane damage occurs in the hopanoid-free versions of *R*. *palustris* [13], *B*. *cenocepacia* [10], *B*. *multivorans* [11,12], and *Bradyrhizobium* [14]. This prompted speculation that hopanoids play a role in membrane permeability to cations and/or protons [13]. The hypothesis that hopanoids play a role in membrane permeability has a long history [6,9], and our results are consistent with this idea. Other work has demonstrated that hopanoids are localized to the outer membrane, where they interact with peptidoglycan and play a role in cell division [38]. The increased toxicity of a broad range of compounds in the absence of hopanoids suggests that hopanoids play a role in modulating toxicity, and perhaps hopanoid-free mutants render cells prone to leakiness or other inability to deal with toxins. Nevertheless, the peptidoglycan biosynthesis inhibitor ampicillin does not produce significantly different zones of inhibition in these two strains, suggesting that the potential barrier role of hopanoids is not involved in resistance to ampicillin. Recent data imply that diplopterol may function in modulating the membrane ordering of saturated fatty acids [46], a suggestion supported by observations that hopanoids localize to particular cellular locations in some strains [47]. Saturated fatty acids comprise less than 10% of the membrane lipids in *Methylobacterium*, but saturated moieties are also present in the lipid A present in the outer membrane, which may also be modulated by diplopterol [46]. This supports previously suggested [46] interactions between hopanoids and lipid A.

Although we have thus far treated hopanoids as a single category, the diverse structures of hopanoids may play different physiological roles in bacteria. In *M*. *extorquens* AM1, approximately half the hopanoid product is diplopterol, which is the simplest amphiphilic hopanoid [4]. Adenosylhopane is not accumulated, but is an intermediate in hopanoid synthesis which is converted to bacteriohopanetetrol cyclitol ether (BHT-CE; structure VI), and guanidine-substituted BHT-CE (structure VII) [20]. Yet other bacteria, such as *Rhodopseudomonas* and *Nitrosomonas*, accumulate adenosylhopane under some conditions [30]. It remains unresolved whether these are biosynthetic by-products or whether this biosynthetic pathway is tightly regulated by the bacterium to produce a specific suite of products, each with different function. Recent work on expressing hopanoid genes in a cell-free system derived from *Methylobacterium organophilum* has begun to shed light on the function of these genes [48]. The wide range of hopanoid structures suggests the possibility that they serve a wide range of functions; this is consistent with biophysical evidence suggesting a diversity of functional roles [49].

Complementation by providing *shc* via a plasmid or as a chromosomal replacement resulted in a full return to wild-type for several phenotypes, such as lipid composition, but only partial restoration for growth rate. These results suggested that the original 41C5 isolate with the *shc*::miniTn*5* allele contained an additional compensatory mutation. Accordingly, we identified an additional mutation relative to WT DM4. This was in a sulfonate transporter, which is consistent with hopanoids functioning in membranes. It is currently unclear whether the synonymous mutation identified affects expression of this gene, but there is precedent for single synonymous mutations to result in large changes in expression and fitness in other metabolic pathways in *M*. *extorquens* [50]. Future work will be required to determine whether this mutations underlies the lack of complementation, as well as to determine it arose as a suppressor mutations that was selectively advantageous in strains lacking hopanoids. These results may be quite interesting in terms of further uncovering the physiological function of hopanoids.

Our results indicate that hopanoids contribute to the fitness of *M*. *extorquens* DM4 under nearly all tested conditions. We observed a series of growth defects for the *shc* mutant beyond what had been previously tested, including extreme flocculation that appears to be related to cellulose biosynthesis. Consistent with other microbes, the *shc* mutant displayed increased sensitivity to a wide variety of toxic agents. These results confirm an emerging picture of the physiological role of hopanoids as important agents of membrane barrier function, while the cellulose interaction suggests that the specific functions that are dependent on hopanoids may differ among organisms.

In many other hopanoid-containing bacteria in which hopanoid-free mutants have been examined, growth defects have been seen under extremes of pH, temperature, and when subjected to stresses such as detergents and antibiotics [4,10–12]. These phenotypes are also present in *Methylobacterium*, which additionally shows growth defects under usual growth temperature and pH. However, hopanoids are not required for growth as in *B*. *diazoefficiens* [15]. The phenotype of hopanoid-free *M*. *extorquens* DM4 offers one more example to the growing set of model organisms showing membrane stress in the absence of hopanoids. Understanding the important role of hopanoids may inspire new hypotheses to understand how most bacteria have adapted to life without hopanoids [51].

## Supporting information

S1 Fig Structures of hopanoids and hopanoid precursor in *M*. *extorquens* DM4. I) diploptene, II) diplopterol, III) methyldiplopterol, IV) C_32_ hopanol V) bacteriohopanetetrol, VI) bacteriohopanetetrol cyclitol ether, VII) guanidine-substituted bacteriohopanetetrol cyclitol ether, VIII) tetrahymanol, IX) squalene.
(PDF)
