# Supplementary figures and images for: Hopanoid-free Methylobacterium extorquens DM4 overproduces carotenoids and has widespread growth impairment
Source: PLoS One. 2017 Mar 20;12(3):e0173323. doi: 10.1371/journal.pone.0173323 (PMC5358736; doi:10.1371/journal.pone.0173323)

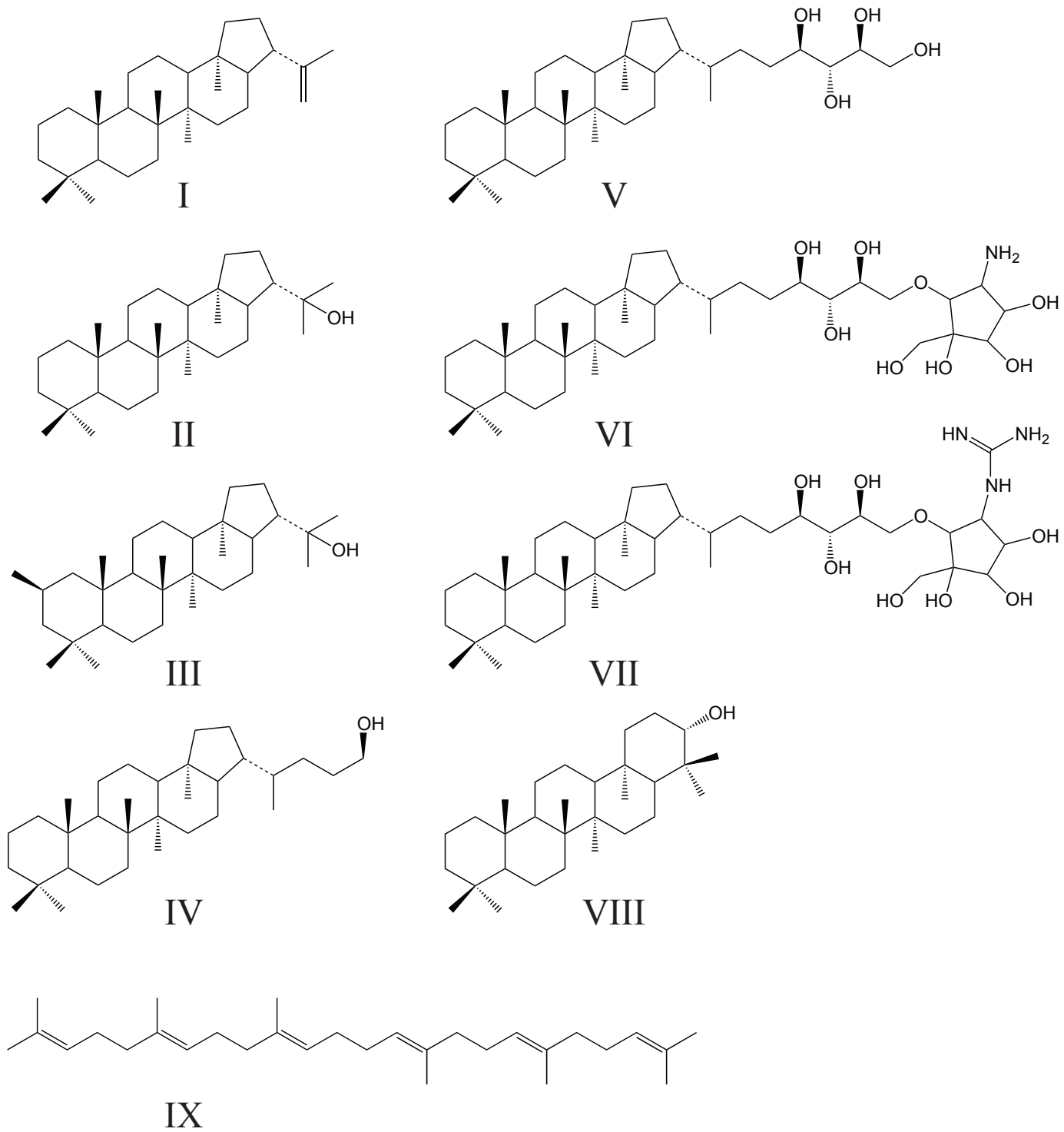

S1 Fig

Supplement: S1 Fig — I) diploptene, II) diplopterol, III) methyldiplopterol, IV) C32 hopanol V) bacteriohopanetetrol, VI) bacteriohopanetetrol cyclitol ether, VII) guanidine-substituted bacteriohopanetetrol cyclitol ether, VIII) tetrahymanol, IX) squalene. (PDF) [file pone.0173323.s001.pdf]
